# Supplementary material for: Fidelity and adherence to a liquefied petroleum gas stove and fuel intervention: the multi-country Household Air Pollution Intervention Network (HAPIN) trial
Source: medRxiv. 2023 Jun 27:2023.06.20.23291670. Preprint. [Version 1] doi: 10.1101/2023.06.20.23291670 (PMC10327189; doi:10.1101/2023.06.20.23291670)
Supplement: Supplement 1 [file NIHPP2023.06.20.23291670v1-supplement-1.pdf]

Supplemental Materials

Figure S1. Number of participants receiving a stove repair out of all enrolled participants by month. Dashed line indicates onset of the global COVID-19 pandemic (March 17, 2020).

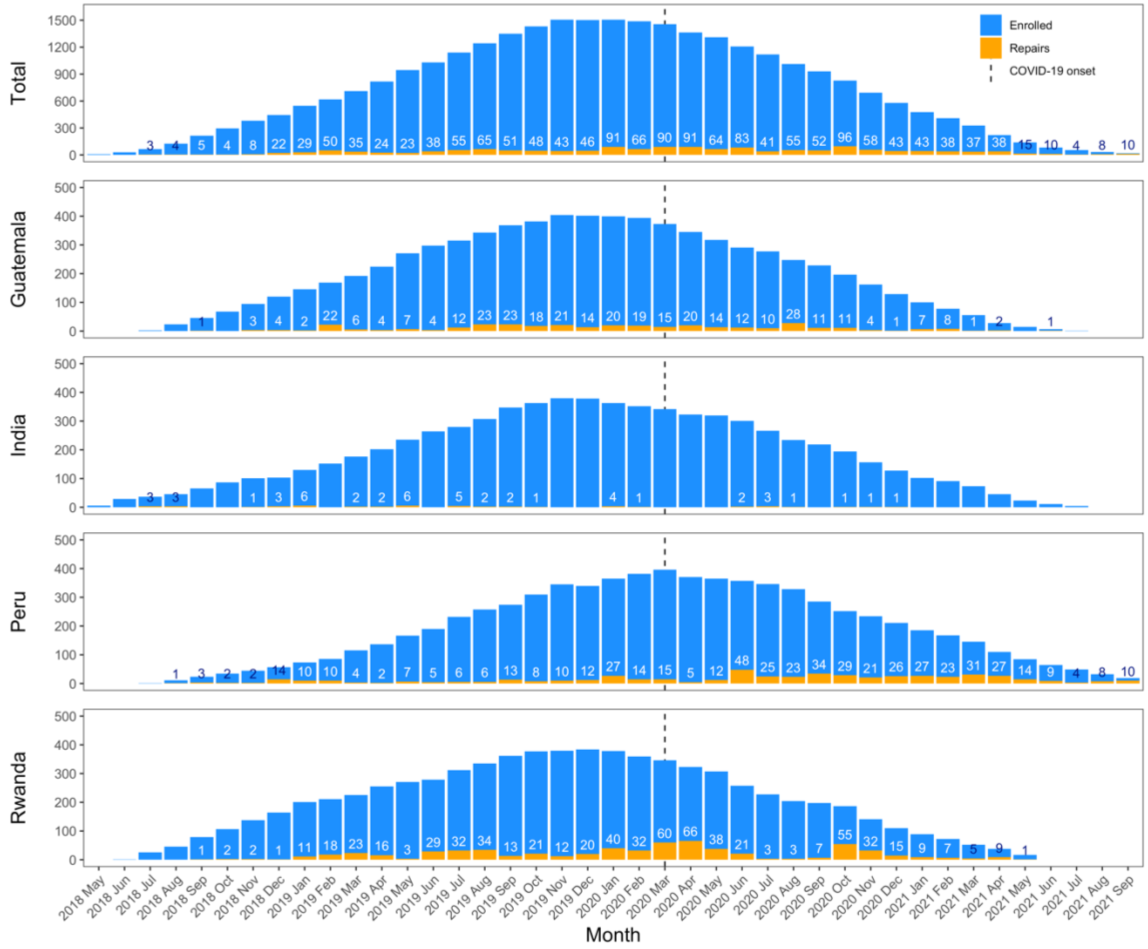

Figure S2. Frequency of the percent of stove-use-monitored days in which traditional stove use (TSU) was detected via stove use monitors (SUMs) in intervention households during the pregnancy period.

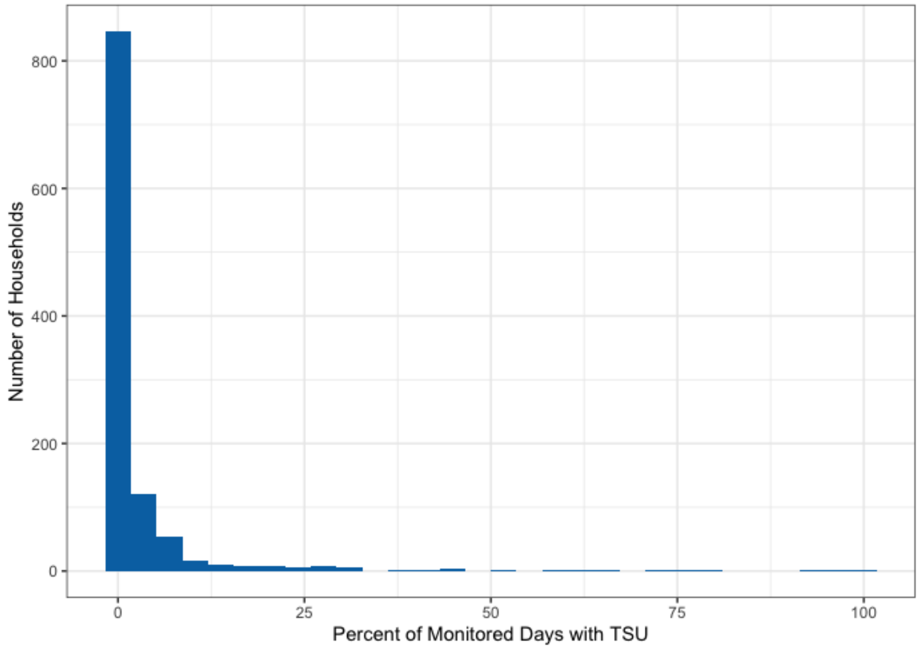

Figure S3. Frequency of the percent of stove-use-monitored days in which traditional stove use (TSU) was detected via stove use monitors (SUMs) in intervention households during the post-birth or infancy period.

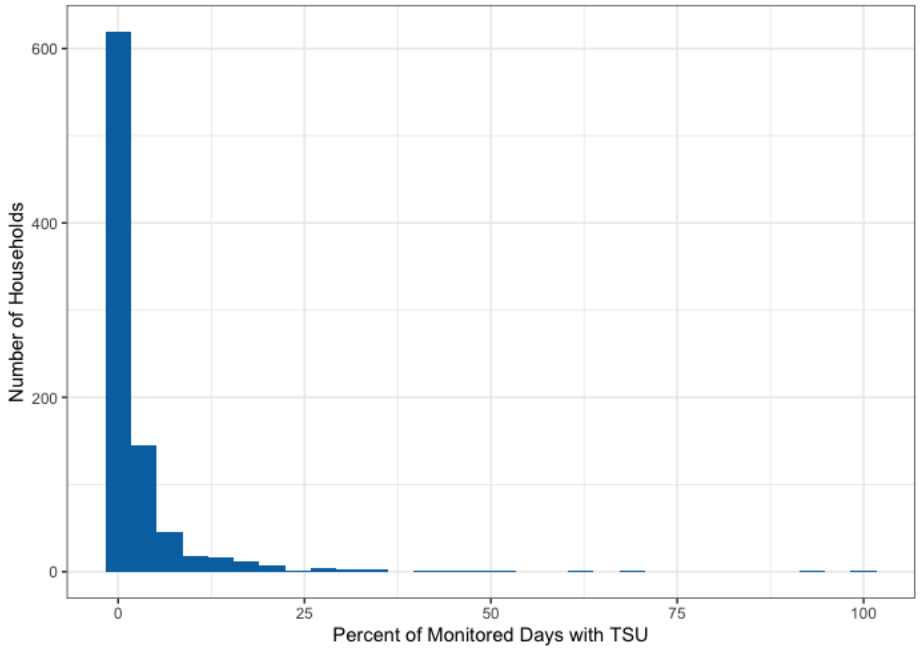

Table S1. Reports of general problems or concerns with the LPG stove and running out of LPG at behavioral reinforcement visits in pregnancy ("preg"), the post-birth or infancy period ("infcy"), and total across the full trial ("full") among intervention participants.

	Guatemala			India			Peru			Rwanda			Total		
Study period in relation to the baby's birth	Preg	Infcy	Full	Preg	Infcy	Full	Preg	Infcy	Full	Preg	Infcy	Full	Preg	Infcy	Full
N Intervention Participants	400	382	400	398	384	398	394	379	394	392	372	392	1584	1517	1584
# (%) of intervention participants who indicated a problem or concern about LPG use at any reinforcement visit	58 (15%)	145 (38%)	169 (42%)	3 (1%)	0	3 (1%)	146 (37%)	140 (37%)	211 (54%)	68 (17%)	291 (78%)	302 (77%)	275 (17%)	576 (38%)	685 (43%)
Median (Q1, Q3) # of reinforcement visits in which participant indicated a problem or concern per participant, among those with >=1 problems/ concerns	1 (1, 1)	1 (1, 2)	1 (1, 2)	1 (1, 1)	0	1 (1, 1)	1 (1, 2)	2 (1, 3)	2 (1, 3)	1 (1, 1)	2 (1, 3)	2 (1, 3)	1 (1, 2)	2 (1, 3)	2 (1, 3)

# (%) of intervention participants who reported running out of LPG at any reinforcement visit	4 (1%)	39 (10%)	42 (11%)	0	0	0	27 (7%)	91 (24%)	105 (27%)	40 (10%)	255 (69%)	263 (67%)	71 (4%)	385 (25%)	410 (26%)
Median # (Q1, Q3) of reinforcement visits in which participant reported running out of LPG per participant, among those with >=1 instance of running out	1.0 (1, 1.5)	1.0 (1, 1)	1.0 (1, 1)	0	0	0	1.0 (1, 1)	1.0 (1, 2)	1.0 (1, 2)	1.0 (1,1)	2.0 (1, 2)	2.0 (1, 2)	1.0 (1, 1)	1.0 (1, 2)	1.0 (1, 2)

Table S2. Types of repairs made to LPG equipment by country and overall.

	Guatemala	India	Peru	Rwanda	Total
Total repair visits made	348	50	547	641	1586
Types of repairs					
Stove*	208 (60%)	32 (64%)	443 (81%)	611 (95%)	1294 (82%)
Knobs/burners	23 (7%)	23 (46%)	309 (56%)	513 (80%)	868 (55%)
Stove valves	187 (54%)	7 (14%)	113 (21%)	4 (1%)	311 (20%)
Stove or part replaced	1 (0.3%)	3 (6%)	41 (8%)	90 (14%)	135 (9%)
LPG cylinder/regulator	3 (1%)	13 (26%)	54 (10%)	13 (2%)	83 (5%)
Hose and connectors	1 (0.3%)	7 (14%)	127 (23%)	8 (1%)	143 (9%)
Switch valve	139 (40%)	N/A	N/A	12 (2%)	151 (15%)

*Only includes most commonly reported stove problems; a few "other" responses not summarized here
NOTE: Numbers may add up to greater than 100% because multiple problems may have been fixed at the same visit.

Repairs to the stove were most common, with repairs to the knobs or burners being most frequent in India, Peru, and Rwanda and repairs to the stove valves most frequent in Guatemala. Repairs to the switch valve (which was only used in Guatemala and Rwanda, to make it easier for households to switch from one cylinder of LPG when it became empty to the other one in the household) were much more common in Guatemala than in Rwanda. Repairs to the LPG cylinder or regulator were most common in India, and repairs to the hose connecting the cylinder to the stove were most common in Peru. Most repairs were completed on the same day as identification or request; the median (Q1, Q3) number of days between identification or request for a repair and successful completion of the repair was 0 (0, 0). Among the 1,563 intervention participants in which observation of the LPG stove was done at the conclusion of their participation in the trial, most LPG stoves were observed to be functioning properly (98.5%). Of the 23 stoves that were not functioning properly, the main two burners were still functioning in 19 (83%), and the 4 with non-working burners were repaired prior to the participant's exit from the study (data not shown).

Table S3. Observations of traditional stove use and follow-up behavioral reinforcement in pregnancy ("preg"), the post-birth or infancy period ("infcy"), and total across the full trial ("full") among intervention participants.

	Guatemala			India			Peru			Rwanda			Total		
Study period in relation to the baby's birth	Preg	Infcy	Full	Preg	Infcy	Full	Preg	Infcy	Full	Preg	Infcy	Full	Preg	Infcy	Full
Total observations made	7012	19276	26288	2766	7079	9845	3360	8086	11446	2571	5091	7662	15709	39532	55241
n (%) of observation visits with traditional stove use observed	135 (2%)	413 (2%)	548 (2%)	3 (0.1%)	2 (0.03%)	5 (0.1%)	173 (5%)	347 (4%)	520 (5%)	131 (5%)	302 (6%)	433 (6%)	442 (3%)	1064 (3%)	1506 (3%)
n (%) of traditional stoves that were observed to be recently used with no SUMs installed	97 (72%)	309 (75%)	406 (74%)	0	1 (50%)	1 (20%)	17 (10%)	41 (12%)	58 (11%)	13 (10%)	57 (19%)	70 (16%)	127 (29%)	408 (38%)	535 (36%)
n (%) of traditional stove use observations with a	-	-	420 (77%)	-	-	2 (40%)	-	-	514 (99%)	-	-	410 (95%)	-	-	1346 (89%)

reinforcement visit at a later date															
Median (Q1, Q3) days between traditional stove use observation and reinforcement visit	-	-	26.5 (10, 179.5)	-	-	33.5 (3, 64)	-	-	0 (0, 0)	-	-	19 (0, 72)	-	-	9 (0, 56)

Table S4. Stove use monitoring in intervention households by country.

	Guatemala	India	Peru	Rwanda	Total
**# Intervention households who received LPG stove**	**400**	**398**	**394**	**392**	**1584**
# Households who voluntarily removed TS at LPG delivery and did not re-install it during pregnancy (%)	265 (66%)	214 (54%)	8 (2%)	10 (3%)	497 (31%)
# Households missing SUMs data during pregnancy for unknown reason* (%)	2 (0.5%)	2 (0.5%)	1 (0.3%)	43 (11%)	48 (3%)
**Total households with valid SUMs during pregnancy**	**133**	**182**	**385**	**339**	**1039**
# Households with miscarriage or stillbirth	16	10	9	13	48
# Households with infant death or drop-out <14 days after birth	2	4	6	7	19
**# live births with post-birth follow-up of mother >14 days**	**382**	**384**	**379**	**372**	**1517**
# Households who removed TS at LPG delivery and did not re-install it during pregnancy or post-birth (%)	222 (58%)	204 (53%)	5 (1%)	5 (1%)	436 (29%)
# Households who removed TS during pregnancy or <14 days after birth and did not re-install post-birth (%)	0	28 (7%)	33 (9%)	0	61 (4%)
# Households missing SUMs data post-birth for unknown reason* (%)	58 (15%)	13 (3%)	37 (10%)	35 (9%)	143 (9%)
**Total households with valid SUMs post-birth**	**102**	**139**	**304**	**332**	**877**
**Total households with valid SUMs in pregnancy and/or post-birth periods**	**164**	**184**	**387**	**360**	**1095**

TS=Traditional stove; SUMs=Stove use monitors; LPG=Liquefied petroleum gas
*Reasons for missing SUMs data may include SUM device errors, participant manipulation or removal of the SUMs, participant misclassification (i.e., failure to record removal of a traditional stove, or mistakenly indicating that participant retained a traditional stove when they had removed it), fieldworker errors in SUM installation, inability to match SUMs data to a household, among others.

Table S5. Additional details on traditional stove monitoring and traditional stove use (TSU) based on SUMs data from intervention households in pregnancy (“preg”), the post-birth or infancy period (“infcy”), and total across the full trial (“full”). This table complements the data in Table 6 of the main paper.

	Guatemala			India			Peru			Rwanda			Total		
Study period in relation to the baby’s birth	Preg	Infcy	Full	Preg	Infcy	Full	Preg	Infcy	Full	Preg	Infcy	Full	Preg	Infcy	Full
Total households with valid SUMs data	133	102	164	182	139	184	385	304	387	339	332	360	1039	877	1095
Days with stove-use-monitoring per household: median (Q1, Q3)	99 (29, 146)	358 (153.2, 392.8)	169.5 (41, 501.2)	127 (91.2, 149.5)	366 (315.5, 376)	460 (156.5, 510)	145 (121, 170)	370 (205.2, 384)	471 (196.5, 538)	134 (95, 161)	338 (243.8, 387)	451.5 (327.8, 511.2)	134 (97.5, 161)	363 (234, 385)	435 (193, 521)
Percent of monitored days with TSU detected: mean (range)	3.8 (0 - 95.2)	6.8 (0 - 100)	4.6 (0 - 94)	1.5 (0 - 53.2)	0.3 (0 - 6.1)	1.1 (0 - 53.2)	4.3 (0 - 81)	3.6 (0 - 51.8)	4.3 (0 - 81)	1.8 (0 - 94.1)	2.1 (0 - 41.6)	1.9 (0 - 31.5)	2.9 (0 - 95.2)	2.9 (0 - 100)	3.0 (0 - 94)
Households with no SUM-detected TSU: N (%)	105 (78.9%)	44 (43.1%)	96 (58.5%)	143 (78.6%)	113 (81.3%)	129 (70.1%)	177 (46%)	145 (47.7%)	126 (32.6%)	194 (57.2%)	114 (34.3%)	106 (29.4%)	619 (59.6%)	416 (47.4%)	457 (41.7%)

Households with < 1 day with TSU per 30 days of monitoring	117 (88.0 %)	72 (70.6 %)	133 (81.1 %)	167 (91.8 %)	135 (97.1 %)	172 (93.5 %)	288 (74.8 %)	228 (75%)	273 (70.5%)	293 (86.4 %)	274 (82.5 %)	307 (85.3 %)	865 (83.3 %)	709 (80.8 %)	885 (80.8%)

Table S6. Traditional stove use (TSU) based on SUMs data from the subset of intervention households with an enrolled non-pregnant adult woman (40-79 years old) across the full trial.

	Guatemala	India	Peru	Rwanda	Total
**# Intervention households with an enrolled non-pregnant adult woman and SUMs monitoring**	**39**	**28**	**62**	**14**	**143**
Days with stove-use-monitoring per household: median (Q1, Q3)	223.0 (73.0, 526.5)	459.0 (296.2, 531.5)	509.0 (306.8, 538.5)	335.0 (280.0, 464.5)	465.0 (206.0, 533.0)
Proportion of follow-up time monitored by SUMs: median (Q1, Q3)	49.1 (18.8. 96.4)	98.4 (62.4, 99.8)	97.7 (65.2, 99.8)	59.0 (50.8, 86.5)	92.7 (43.9, 99.8)
Percent of monitored days with TSU detected: median (Q1, Q3)	0.3 (0.0, 10.3)	0.0 (0.0, 0.5)	1.3 (0.2, 4.9)	1.0 (0.0, 2.7)	0.4 (0.0, 3.6)
Households with no SUM-detected TSU: N (%)	17 (43.6%)	18 (64.3%)	13 (21.0%)	5 (35.7%)	53 (37.1%)
Avg # days with TSU per 30 days of monitoring	0.1 (0.0. 3.1)	0.0 (0.0, 0.1)	0.4 (0.1, 1.5)	0.3 (0.0, 0.8)	0.1 (0.0, 1.1)
Households with < 1 day with TSU per 30 days of monitoring	24 (61.5%)	27 (96 4%)	42 (67.7%)	12 (85.7%)	105 (73.4%)

Table S7. Traditional stove use based on SUMs data from intervention households pre-COVID-19 and post-COVID-19.

	Guatemala		India		Peru		Rwanda		Total	
	Pre-COVID	Post-COVID	Pre-COVID	Post-COVID	Pre-COVID	Post-COVID	Pre-COVID	Post-COVID	Pre-COVID	Post-COVID
Total households with valid SUMs data	163	74	184	99	383	258	357	226	1087	657
Days with stove-use-monitoring per household: median (Q1, Q3)	89 (38, 302)	132 (72.2, 220.5)	247.5 (121, 349.2)	196.0 (114.5, 263)	181 (90, 300.5)	218.5 (133.2, 354.8)	292 (192, 399)	162 (89.2, 239.5)	225 (110.5, 350)	188 (108, 279)
Proportion of follow-up time monitored by SUMs	30.5 (11.9, 95.9)	98.5 (94.2, 99.3)	96.8 (44.7, 100)	99.2 (97.5, 99.6)	100 (72.5, 100)	99.2 (65.9, 99.6)	91.4 (71.7 100)	89.3 (65.3, 99.2)	95.9 (52.4, 100)	98.7 (72.9, 99.5)
Percent of monitored days with TSU detected: median (Q1, Q3)	0.0 (0.0, 0.9)	0.0 (0.0, 4.2)	0.0 (0.0, 0.0)	0.0 (0.0, 0.0)	0.2 (0.0, 2.3)	0.7 (0.0, 5.5)	0.4 (0.0, 1.6)	0.7 (0.0, 3.5)	0.0 (0.0, 1.6)	0.0 (0.0, 3.4)
Households with no SUM-detected TSU: N (%)	106 (65%)	39 (52.7%)	140 (76.1%)	82 (82.8%)	190 (49.6%)	110 (42.6%%)	144 (40.3%)	98 (43.4%)	580 (53.4%)	329 (50.1%)
Avg # days with TSU per 30 days of monitoring	0.0 (0.0, 0.3)	0.0 (0.0, 1.3)	0.0 (0.0, 0.0)	0.0 (0.0, 0.0)	0.1 (0.0, 0.7)	0.2 (0.0, 1.6)	0.1 (0.0, 0.5)	0.2 (0.0, 1.1)	0.0 (0.0, 0.5)	0.0 (0.0, 1.0)
Households with < 1 day with TSU per 30 days of monitoring	135 (82.8%)	52 (70.3%)	171 (92.9%)	98 (99%)	303 (79.1%)	175 (67.8%)	315 (88.2%)	164 (72.6%)	924 (85%)	489 (74.4%)

Table S8. Traditional stove use based on SUMs data from intervention households in early-COVID-19 (March 17, 2020 – July 17, 2020) and late-COVID-19 (after July 17, 2020) periods.

	Guatemala		India		Peru		Rwanda		Total	
	Early-COVID	Late-COVID	Early-COVID	Late-COVID	Early-COVID	Late-COVID	Early-COVID	Late-COVID	Early-COVID	Late-COVID
Total households with valid SUMs data	74	35	99	63	258	192	225	146	656	436
Days with stove-use-monitoring per household: median (Q1, Q3)	120.5 (72.2, 122)	118 (71, 196)	122 (114.5, 122)	122 (81, 206.5)	122 (118.2, 122)	146 (69, 279.8)	120 (77, 122)	104 (62, 188)	122 (99, 122)	124 (69.8, 224)
Proportion of follow-up time monitored by SUMs	98.8 (59.2, 100)	98.4 (92.1, 99)	100 (93.9, 100)	98.9 (98.5, 99.4)	100 (96.9, 100)	98.8 (93.5, 99.5)	98.4 (63.1, 100)	83.9 (51.2, 99)	100 (81.1, 100)	98.5 (77, 99.3)
Percent of monitored days with TSU detected: median (Q1, Q3)	0.0 (0.0, 3.4)	0.0 (0.0, 1.9)	0.0 (0.0, 0.0)	0.0 (0.0, 0.0)	0.0 (0.0, 7.4)	0.0 (0.0, 2.7)	0.0 (0.0, 4.2)	0.0 (0.0, 1.5)	0.0 (0.0, 3.7)	0.0 (0.0, 1.5)
Households with no SUM-detected TSU: N (%)	48 (64.9%)	22 (62.9%)	86 (86.9%)	57 (90.5%)	136 (52.7%)	112 (58.3%)	113 (50.2%)	95 (65.1%)	383 (58.4%)	286 (65.6%)
Avg # days with TSU per 30 days of monitoring	0.0 (0.0, 1)	0.0 (0.0, 0.6)	0.0 (0.0, 0.0)	0.0 (0.0, 0.0)	0.0 (0.0, 2.2)	0.0 (0.0, 0.8)	0.0 (0.0, 1.3)	0.0 (0.0, 0.5)	0.0 (0.0, 1.1)	0.0 (0.0, 0.4)
Households with < 1 day with TSU per 30 days of monitoring	55 (74.3%)	29 (82.9%)	96 (97%)	62 (98.4%)	175 (67.8%)	151 (78.6%)	163 (72.4%)	125 (85.6%)	489 (74.5%)	367 (84.2%)

Table S9. Percent of intervention participants with observations of traditional stove use during pregnancy (“preg”), post-birth or infancy (“infcy”), and total across the full trial (“full”), and extent to which traditional stove use was observed per participant.

	Guatemala			India			Peru			Rwanda			Total		
Study period in relation to the baby’s birth	Preg	Infcy	Full	Preg	Infcy	Full	Preg	Infcy	Full	Preg	Infcy	Full	Preg	Infcy	Full
N intervention participants	400	382	400	398	384	398	394	379	394	392	372	392	1584	1517	1584
n (%) of intervention participants who received an observation visit	399 (99.8 %)	382 (100 %)	399 (99.8 %)	396 (99.5 %)	384 (100 %)	396 (99.5 %)	393 (99.8 %)	379 (100 %)	393 (99.8 %)	375 (95.7 %)	369 (99.2 %)	387 (98.7 %)	1563 (98.7 %)	1514 (99.8 %)	1575 (99.4 %)
Median (Q1, Q3) # of observation visits, out of all intervention	17 (13, 21)	49.5 (39, 60)	65 (52, 80)	7 (6, 8)	19 (17, 21)	26 (22, 29)	9 (7, 10)	22 (20, 24)	30 (28, 32)	7 (4, 9)	14 (9, 19)	22 (14, 26)	9 (6, 11)	21 (17, 26)	29 (23, 34)

participants															
n (%) of intervention participants with any observation of traditional stove use (out of those with any observation visit)	71 (18%)	204 (53%)	224 (56%)	3 (0.8%)	2 (0.5%)	5 (1.3%)	112 (29%)	150 (40%)	202 (51%)	85 (23%)	193 (52%)	229 (58%)	271 (17%)	549 (36%)	660 (42%)
Median (Q1, Q3) # of traditional stove use observations per participant, out of those with any TSU observations	1.0 (1, 2)	1.0 (1, 2)	1.0 (1, 2)	1.0 (1, 1)	1.0 (1, 1)	1.0 (1, 1)	1.0 (1, 2)	2.0 (1, 3)	2.0 (1, 3)	1.0 (1, 2)	1.0 (1, 2)	1.0 (1, 2)	1.0 (1, 2)	1.0 (1, 2)	2.0 (1, 3)

Median (Q1, Q3) % of observations with TSU observed (among those with any TSU observations)	6.5% (4.5, 11.1)	2.5% (1.9, 4.1)	2.1% (1.5, 3.7)	14.3 % (14.3, 16.7)	8.4% (4.3, 12.5)	3.7% (3.4, 3.7)	14.3 % (11.1, 26.1)	8.3% (4.8, 14.3)	6.5% (3.3, 11.5)	16.7 % (12.5, 28.6)	9.1% (5.9, 14.3)	7.1% (4.3, 11.8)	12.5 % (9.1, 25.0)	5.4% (3.3, 11.1)	4.5% (3.0, 9.1)

Table S10. Observations of traditional stove use in intervention households missing SUMs data.

	Guatemala	India	Peru	Rwanda	Total
**# Intervention households who received LPG stove**	**400**	**398**	**394**	**392**	**1584**
# intervention households with no SUMs monitoring during pregnancy	269 (67%)	216 (54%)	9 (2%)	53 (14%)	547 (35%)
Of those with no SUM in pregnancy, n (%) who received any observation visit during pregnancy	268 (99.6%)	216 (100%)	8 (89%)	39 (74%)	531 (97%)
Median (Q1, Q3) number of observation visits conducted during pregnancy among those with no SUMs and >=1 observation visit completed	16 (13, 20)	7 (5, 8)	5.5 (2.5, 9)	2 (1, 5)	10 (6, 16)
Median (Q1, Q3) # of observations of TSU among those with no SUMs and >=1 observation visit completed	0 (0, 0)	0 (0, 0)	0 (0, 0)	0 (0, 0)	0 (0, 0)
**# Intervention households followed post-birth**	**382**	**384**	**379**	**372**	**1517**
# intervention households with no SUMs monitoring post-birth	280 (73%)	245 (64%)	75 (20%)	40 (11%)	640 (42%)
Of those with no SUM post-birth, n (%) who received any observation visit post-birth	280 (100%)	245 (100%)	75 (100%)	39 (98%)	639 (99.8%)
Median (Q1, Q3) number of observation visits conducted among those with no SUMs and >=1 observation visit completed	49 (39, 61)	19 (17, 21)	20 (18, 22)	4 (3, 8)	22 (18, 47)
Median (Q1, Q3) observations of TSU among those with no SUMs and >=1 observation visit completed	0 (0, 1)	0 (0, 0)	0 (0, 0)	0 (0, 1)	0 (0, 0)

Table S11. Percent of intervention and control participants who ever moved during the trial, including use of biomass by intervention participants during moves and use of clean fuels by control participants during moves.

	Guatemala		India		Peru		Rwanda		Total	
	Intvn	Cntrl	Intvn	Cntrl	Intvn	Cntrl	Intvn	Cntrl	Intvn	Cntrl
n	400	400	398	399	394	402	392	404	1584	1605
# (%) households that ever moved	65 (16%)	77 (19%)	96 (24%)	98 (25%)	116 (29%)	131 (33%)	71 (18%)	60 (15%)	348 (22%)	366 (23%)
# (%) Intervention participants who moved to house where biomass is used at all	13 (3%)	---	52 (13%)	---	10 (3%)	---	4 (1%)	---	79 (5%)	---
Median (Q1, Q3) days spent by intervention participants in house where biomass is used at all	104 (83, 121)	---	153 (86.5, 223.5)	---	86.5 (26, 168)	---	94 (64, 122)	---	136 (71, 215)	---
# (%) Control participants who moved to house where clean fuel is used exclusively	---	2 (0.5%)	---	7 (2%)	---	42 (10%)	---	0	---	51 (3%)
Median (Q1, Q3) days spent by control participants in house where clean fuel is used exclusively	---	506 (471, 540)	---	147 (18, 309)	---	136 (84, 220)	---	0	---	141 (79, 246)

The most frequent reasons for moves included visiting the mother's or mother-in-law's house, moving to a new personal residence, and moving to a seasonal residence. Visiting the mother's or mother-in-law's house was the most common reason for moves in Guatemala, India, and Peru, while moving to a new personal residence was most common in Rwanda. Reasons were similar between intervention and control participants. Across countries, control participants who moved to a house where clean fuel was used exclusively spent a median (Q1, Q3) of 141 (79, 246) days in the new or temporary home. Intervention participants who moved to a home where biomass is used at all spent a median (Q1, Q3) of 136 (71, 215) days in the new or temporary home, but only 29% (n=23) reported being the primary cook in that home.
